# First preclinical SPECT/CT imaging and biodistribution of [^165^Er]ErCl_3_ and [^165^Er]Er-PSMA-617

**DOI:** 10.1186/s41181-024-00312-9

**Published:** 2024-12-18

**Authors:** Behrad Saeedi Saghez, Cristina Rodríguez-Rodríguez, Pedro Luis Esquinas, Helen Merkens, François Bénard, Valery Radchenko, Hua Yang

**Affiliations:** 1 Life Sciences Division, TRIUMF, 4004 Wesbrook Mall, Vancouver, BC V6T 2A3 Canada; 2 Department of Chemistry, University of British Columbia, 2036 Main Mall, Vancouver, BC V6T 1Z1 Canada; 3 Department of Physics and Astronomy, University of British Columbia, 6224 Agricultural Road, Vancouver, BC V6T 1Z1 Canada; 4 Faculty of Pharmaceutical Sciences, University of British Columbia, 2405 Wesbrook Mall, Vancouver, BC V6T 1Z3 Canada; 5 Department of Integrative Oncology, BC Cancer Research Institute, 675 West 10th Avenue, Vancouver, BC V5Z 1L3 Canada; 6 Department of Molecular Oncology, BC Cancer Research Institute, 675 West 10th Avenue, Vancouver, BC V5Z 1L3 Canada; 7 Department of Radiology, University of British Columbia, 2775 Laurel Street, Vancouver, BC V5Z 1M9 Canada; 8 Department of Chemistry, Simon Fraser University, 8888 University Drive, Burnaby, BC V5A 1S6 Canada

**Keywords:** ^165^Er, Erbium-165, Auger electron emitter, Phantom imaging, SPECT imaging, Preclinical study, PSMA-617

## Abstract

**Background:**

^165^Er (t_1/2_ = 10.4 h, E_x-ray_ = 47.1 keV (59.4%) and 54.3 keV (14.3%)) is a promising radionuclide suitable for targeted Auger electron therapy of cancer. ^165^Er can be produced at a relatively low cost, high yield, and high purity using small medical cyclotrons. As a late lanthanide, ^165^Er is easy to label and can be used as a surrogate for other lanthanides or Ac in proof-of-concept studies. In this report, we explore the radiochemistry, in vitro, and in vivo behavior of [^165^Er]ErCl_3_ and [^165^Er]Er-PSMA-617 to showcase the application of this radionuclide. Particularly, we report the first phantom and preclinical SPECT imaging of this radionuclide leveraging its characteristic X-ray photon emissions.

**Results:**

The ^165^Ho(p,n)^165^Er nuclear reaction using a 13 MeV cyclotron demonstrated production yields of up to 25 ± 5 MBq. µA^−1^ h^−1^ at the end of the bombardment. After the purification (4.0 ± 0.5 h) using a sequential combination of cation exchange and extraction chromatography, 4-h irradiation produced up to 1.5 GBq of [^165^Er]ErCl_3_. High molar activity [^165^Er]Er-PSMA-617 was prepared (~ 200 MBq/nmol). [^165^Er]Er-PSMA-617 showed a Log*D*_7.4_ value of -2.34 ± 0.24 meaning high hydrophilicity of the complex as expected. The stability of [^165^Er]Er-PSMA-617 in saline, human, and mouse serum was studied and showed intact tracer after 12 h in all three cases. [^165^Er]ErCl_3_ and [^165^Er]Er-PSMA-617 were both taken up by LNCaP cells. PSMA-617 has IC_50_ at nanomolar range for [^165^Er]Er-PSMA-617 in LNCaP cells. SPECT images with preclinical phantoms showed good uniformity, spatial resolution, and quantitative accuracy. SPECT/CT imaging in LNCaP tumor-bearing mice injected with [^165^Er]Er-PSMA-617 showed high tumor uptake and quantitative accuracy when comparing the results to ex vivo biodistribution %IA/g values. Mice injected with [^165^Er]ErCl_3_ showed uptake in bone structures and excretion through both liver and kidneys.

**Conclusions:**

This study demonstrated the preclinical use of ^165^Er for the first time. Using [^165^Er]ErCl_3_ and [^165^Er]Er-PSMA-617 as examples, the radiochemistry, cell, and animal studies showed that ^165^Er can be used as a tool for evaluating targeted radiopharmaceuticals. The X-ray emission from ^165^Er can be used for quantitative SPECT imaging in mice.

**Supplementary Information:**

The online version contains supplementary material available at 10.1186/s41181-024-00312-9.

## Background

Erbium-165 (^165^Er, t_1/2_ = 10.4 h) is a pure Auger electron-emitting radionuclide (Bolcaen et al. 2023) that decays 100% through electron capture (NNDC 2024). It is an interesting radionuclide for studying the radiobiological effect of Auger emissions because it does not emit other particles. ^165^Er also emits high-intensity X-rays (E_x-ray_ = 47.1 keV (59.4%) and 54.3 keV (14.3%)) (NNDC 2024). In this report, we wanted to explore the preclinical use of ^165^Er and if the X-ray emissions could be used for single photon emission computed tomography (SPECT) imaging. The imaging capability will make this radionuclide more interesting – it would allow ^165^Er to be a standalone theranostic reagent, or to pair with β^−^ emitter ^169^Er (t_1/2_ = 9.39 days) for theranostics (NNDC 2024). On the chemistry front, as a late lanthanide, erbium is easily coordinated by DOTA and similar chelators. Furthermore, the fact that ^165^Er can be produced at low cost using small medical cyclotrons with high purity and high yield (Gracheva et al. 2020, Silva et al. 2021, and Saeedi Saghez et al. 2024), marks this radionuclide as a useful tool to test chelators and targeting vectors at smaller centers or developing countries.

There have been multiple reports on the production and purification of ^165^Er in the literature. Because natural Ho is monoisotopic and the cross-section is favorable, ^165^Er was produced with low cost, high yield, and high purity at small medial cyclotrons (12–13 MeV) (Gracheva et al. 2020; Silva et al. 2021; Saeedi Saghez et al. 2024). [^165^Er]Er-PSMA-617 synthesis was previously reported and its distribution coefficient (log*D*_7.4_) and stability in human serum was demonstrated (Silva et al. 2021).

The present study aims to explore the application of ^165^Er in preclinical studies. Using the ^165^Er production and purification method we developed, [^165^Er]Er-PSMA-617 was synthesized at high molar activity. Its lipophilicity, and stability in saline, human serum, and mouse serum were studied. The uptake of [^165^Er]ErCl_3_ and [^165^Er]Er-PSMA-617 in LNCaP cells were investigated. The half maximum inhibition concentration (IC_50_) of PSMA-617 for [^165^Er]Er-PSMA-617 was measured using LNCaP cells. The preclinical SPECT imaging capability of ^165^Er was validated and analyzed with both uniformity and resolution phantoms. The biodistribution and quantitative SPECT images of [^165^Er]ErCl_3_ and [^165^Er]Er-PSMA-617 in LNCaP tumor-bearing mice were evaluated. This proof-of-concept study demonstrated the use of ^165^Er for evaluating drug candidates preclinically. Furthermore, this study showed the feasibility of imaging X-ray emissions of ^165^Er using pre-clinical SPECT. To date, to the best of our knowledge, this work is the first report on the biodistribution and SPECT imaging of [^165^Er]ErCl_3_ and [^165^Er]Er-PSMA-617.

## Methods

### General

All solvents and reagents were purchased from commercial suppliers and used without further purification. Ho metal (99.9%-1 ppm natural Er) was purchased from the United States Department of Energy (DOE) Ames Laboratory Materials Preparation Center. The pH was controlled using a pH meter (Orion 118 Star, Thermo Fisher Scientific), calibrated with commercially available buffers (pH 4.00, pH 7.00, and pH 10.00) purchased from Fisher Scientific. PSMA-617 (vipivotide tetraxetan, HY-117410) was purchased from MedChemExpress. LNCaP cell line was purchased from Cedarlane Corporation. Radiolabelling studies were monitored using radio thin-layer chromatography (radio-TLC) with silicic acid (SA)-impregnated paper TLC plates (Agilent technologies). TLC imaging was performed using an AR-2000 imaging scanner (Eckert & Ziegler) equipped with P-10 gas, and radiochemical yields (RCYs) were analyzed using WinScan V3_14 software. Radio-HPLC was carried out using an Agilent 1200 instrument equipped with a Phenomenex Luna C18 reverse phase column (100 × 46 mm, 5 µm) and a GABI star radioactive HPLC flow monitor (Elysia-ray test GmbH). Activity was quantified using a calibrated high purity germanium (HPGe) detector (Mirion Technologies (Canberra) Inc.) with Genie 2000 software and an ionization chamber dose calibrator (CRC-15R, setting #260, Capintec Inc.) calibrated with the HPGe gamma spectrometer. A BioScan shaker and heater for microcentrifuge tubes was used to perform all radiolabelling experiments. A Countess 3 automated cell counter with countess cell counting chamber slides (ThermoFisher Scientific) was used to count the number of cells. An Automatic Gamma Counter (NaI detector) from Hidex was used to measure the activity counts per second (CPS) for cell studies and biodistribution experiments in 30–60 keV region (the CPS was converted to Bq by calibration of gamma counter using known activities of ^165^Er). SPECT and Computed Tomography (CT) studies were performed using a Versatile Emission Computed Tomography (VECTor) micro PET/SPECT/CT imaging system (MILabs, Utrecht, Netherlands). Image analysis was performed using AMIDE (v. 1.0.6) software. Statistical analysis was performed in Microsoft Excel and presented as average ± standard deviation.

### ^165^Er production and detection

^165^Er was produced and quantified using a previously developed method (Saeedi Saghez et al. 2024). In short, ^165^Ho cyclotron targets were prepared by melting 200 mg of high purity ^165^Ho disks in the recess of tantalum backings (recess diameter: 1 cm, backing diameter: 3 cm, backing thickness: 1 ± 0.1 mm) in the furnace (at 1545 °C under argon).

The targets were irradiated at the TR13 cyclotron at TRIUMF using a solid target holder with a quick-release mechanism. The target holder allowed for active cooling of targets by passing cold water over the back of the target continuously during the irradiation period, which prevented overheating of the target. Since a 25 μm aluminum foil was used to separate the target system from the cyclotron vacuum, the 13 MeV protons energy degraded to 12.8 MeV. The targets were irradiated for 3–6 h with a beam current of 30 μA.

The HPGe gamma spectrometer was calibrated with ^133^Ba and ^152^Eu radioactive sources placed in a 20 mL scintillation vial 5 cm from the detector and counted for 24 h. An efficiency curve was created based on the known activity of the radioactive sources and the intensity of X-ray lines in the 0–60 keV region to quantify ^165^Er.

### ^165^Er dissolution and purification

After irradiation, the target was dissolved in 2 mL of 12 M HCl. The HCl was evaporated, and the produced [^165^Er]ErCl_3_ was redissolved in 1 mL of 70 mM alpha-hydroxyisobutyrate (α-HIB, pH 4.70–4.75). After dissolution, the target solution was loaded on a 50W-X8 cation exchange resin (16.9 g, 25 cm long, 1 cm diameter, conditioned with 150 mL of 1 M NH_4_Cl, 150 mL of deionized (DI) water, and 150 mL of 70 mM α-HIB) and eluted with 350 mL of 70 mM α-HIB (pH 4.75 ± 0.05) at 5 mL/min. The first 150 mL of α-HIB was transferred to waste, and the last 200 mL of α-HIB was collected for further purification.

Then, 1.26 mL of 15.9 M HNO_3_ was added to the 200 mL α-HIB elution from cation exchange resin, and the solution was loaded onto a TK212 resin (500 mg, 0.5 cm diameter, conditioned with 10 mL of 1 M HNO_3_ and 25 mL of 0.1 M HNO_3_) at 5 mL/min. The ^165^Ho was eluted with 60 mL of 0.4 M HNO_3_ into waste at 1 mL/min. The ^165^Er was eluted with 10 mL of 1 M HNO_3_ at 1 mL/min and was collected for further purification.

The collected ^165^Er was loaded on a TK211 resin (250 mg, 0.5 cm diameter, conditioned with 10 mL of 1 M HNO_3_) at 5 mL/min. The ^165^Ho was eluted with 17 mL of 1.25 M HNO_3_ into waste at 1 mL/min. The ^165^Er was eluted with 5 mL of 3 M HNO_3_ at 1 mL/min and was collected for further purification.

The collected ^165^Er was loaded on a TK221 resin (100 mg, 0.5 cm diameter, conditioned with 10 mL of 3 M HNO_3_) at 5 mL/min. The resin was washed with 10 mL of 0.1 M HNO_3_ at 1 mL/min to elute transition metals such as iron, copper, nickel, and other contamination that might have accumulated throughout the production and purification process. The final ^165^Er product was eluted in 2 mL of 0.05 M HCl at 1 mL/min.

### ^165^Er activity escalation and radiolabeling with PSMA-617

**Fig. 1:**
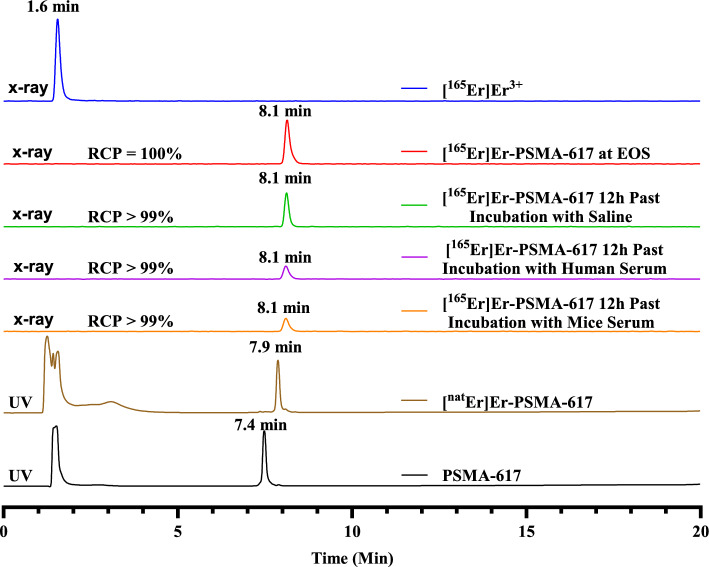
The radio-HPLC gamma traces for [^165^Er]ErCl_3_, [^165^Er]Er-PSMA-617 at EOS, [^165^Er]Er-PSMA-617 after 12 h incubation in saline, human serum, or mice serum along with ultra violet (UV) traces (220 nm) of PSMA-617 and [.^nat^Er]Er-PSMA-617

An increasing amount of ^165^Er activity was reacted with a constant amount of PSMA-617 solution (1 µL of 10^–4^ M, Table 1S in Supplementing Information). The buffer solution used throughout the activity escalation experiments was 2 M ammonium acetate buffer at pH 5.50. 1 M NaOH solution was used to neutralize the 0.05 M HCl solution that contained ^165^Er. The experiments were performed 8 h after the end of bombardment (EoB) with an ^165^Er product (2 mL of 0.05 M HCl), which had an activity concentration of 493 MBq/mL (obtained using 4-h irradiation of a 200 mg Ho target with 30 µA current and 12.8 MeV energy). In all reactions (prepared in duplicates), the amount of PSMA-617 was kept constant (0.1 nmol) while the activity added gradually increased. The reaction mixtures were heated at 90˚C for 10 min under shaking at 600 rounds per minute (rpm). After cooling to room temperature, aliquots of the solutions were spotted on SA impregnated radio-TLC plates and were developed with 50 mM ethylenediaminetetraacetic acid (EDTA) mobile phase at pH 5.50. The TLC plates were analyzed with a radio-TLC plate reader. Under those conditions, unbound ^165^Er moves to the top of the plate (R_f_ > 0.9) while the labeled compound stays close to the bottom of the plate (R_f_ < 0.3) (Fig. 1Sin Supplementing Information).

The experimental molar activity (unpurified) was calculated using Eq. 1:

1 $$MA=\left(\frac{A}{M}\right)\times \left(\frac{RCY}{100}\right)$$

The Eq. 1 used to determine the experimental molar activity of ^165^Er with a specific targeting vector.

In Eq. 1, *MA* is the molar activity of ^165^Er-labelled compound in MBq/nmol of the targeting vector, *A* is the added activity of ^165^Er to the reaction mixture in MBq, *M* is the added number of moles of targeting vector to the reaction mixture in nmol, and *RCY* is the radiochemical yield of the reaction after incubation period obtained using radio-TLC in %.

### [^165^Er]Er-PSMA-617 distribution coefficient

In the distribution coefficient studies, 300 μL of n-octanol and 300 μL of phosphate buffered saline (PBS, pH 7.4) were placed in an Eppendorf tube, 40 μL of [^165^Er]Er-PSMA-617 (10.8 MBq, 0.1 nmol PSMA-617) was added (prepared using similar conditions as Sect. "165Er activity escalation and radiolabeling with PSMA-617"), and the mixture was vortexed for 1 min. The upper and lower phases were separated by centrifugation at 2000 rpm for 30 min. Then, 100 μL of each phase was sampled, and a Hidex gamma counter was used to measure the activity in each sample. Distribution coefficient values were calculated using the Eq. 2:

2 $$Log D_{7.4} = {\text{log}}\;\left( {\frac{{{\text{A}}_{n - octanol} }}{{{\text{A}}_{PBS\;buffer} }}} \right)$$

The Eq. 2 used for the determination of hydrophilicity of [^165^Er]Er-PSMA-617.

In Eq. 2, *log D*_*7.4*_ is the distribution coefficient value, *A*_*PBS buffer*_ is the amount of [^165^Er]Er-PSMA-617 in the aqueous phase in MBq, and *A*_*n-octanol*_ is the amount of [^165^Er]Er-PSMA-617 in n-octanol phase in MBq.

### [^165^Er]Er-PSMA-617 stability in saline and serum

Quality control for the [^165^Er]Er-PSMA-617 radioligand was performed by analytical radio-HPLC, using a Phenomenex Luna C18 reverse phase column (100 × 4.6 mm, 5 µm) and A: 0.1% trifluoracetic acid (TFA) in water, B: 0.1% TFA in acetonitrile; gradient: 90% A/10%B to 100% B over 15 min, flow rate: 1 mL/min. [^165^Er]ErCl_3_, PSMA-617 (10 µL, 10^–3^ M), and [^nat^Er]Er-PSMA-617 were injected into HPLC (in duplicates) to determine the retention time of each of these components before the stability studies.

The stability reactions were prepared in triplicates using ^165^Er product (2 mL of 0.05 M HCl) 10 h after EoB, which had an activity concentration of 308 MBq/mL (obtained using a three hours irradiation of a 200 mg Ho target with 30 µA current and 12.8 MeV energy). Each reaction contained 40 μL of [^165^Er]Er-PSMA-617 (10.8 MBq, 0.1 nmol PSMA-617) prepared using similar conditions as Sect. "165Er activity escalation and radiolabeling with PSMA-617".

Each reaction was mixed at 90 °C for 10 min, followed by analysis with radio-TLC to ensure 100% labeling before incubation with serum or saline. Three reactions were diluted with DI water and injected into HPLC to establish the retention time for [^165^Er]Er-PSMA-617.

The saline stability studies were started by adding 360 µL of saline to each of the three reactions (total volume 402 µL). At 1, 2, 4, and 12 h time points, an aliquot of each reaction was spotted on TLC plates to analyze the labeling with the radio-TLC plate reader. An aliquot was also taken for injection into HPLC (about 500 kBq of activity per injection).

The serum stability studies were started by adding 360 µL of human and mouse serums to each of the six reactions (three reactions per serum, total volume 402 µL). At 1, 2, 4, and 12 h time points, an aliquot of each reaction was spotted on TLC plates to analyze the labeling with the radio-TLC plate reader. Also, 80 µL of each reaction was transferred to another Eppendorf tube at each time point, followed by adding 80 µL of acetonitrile. The solutions were vortexed and centrifuged for 20 min at 14,000 rpm to precipitate serum proteins. The supernatants were transferred to HPLC vials and diluted with water to match the polarity of the initial HPLC eluent (90% water with 10% acetonitrile). An aliquot of each solution was injected into HPLC (100–200 kBq of activity per injection).

### In vitro uptake, internalization, and IC_50_ displacement assays in LNCaP cells

LNCaP cells were grown in Roswell Park Memorial Institute (RPMI) 1640 medium, supplemented with 10% (v/v) fetal bovine serum (FBS) and 1% (v/v) penicillin–streptomycin (PS) in 60 mm plates incubated at 37 °C in 5% CO_2_ environment. The cell media was changed every three to four days, and the cells were passaged at a ratio of 4:10 every week. The number of passages was kept below 10. All radiolabelling were performed at 50 MBq per nmol of PSMA-617 for in vitro studies.

The internalization and uptake of [^165^Er]ErCl_3_ and [^165^Er]Er-PSMA-617 in LNCaP (PSMA +) cells were studied using methods available in the literature (Zhang et al. 2017). Five hundred thousand cells were seeded in each well of a poly-D-lysine coated 24-well plate. Three wells were seeded per each time point, and two time points were tested for a total of 12 experimental wells. The volume of each well was adjusted to 1 mL by adding fresh media, and empty wells were filled with 1 mL of PBS. The 24-well plates were incubated in a 5% CO_2_ environment at 37 °C for 48 h.

At the time of the experiment, the media from each well of the poly-D-lysine coated plate was removed and replaced with 25 kBq of [^165^Er]ErCl_3_ or [^165^Er]Er-PSMA-617 in a solution of 20 mM HEPES and 2 mg/mL bovine serum albumin in PBS (0.4 mL) and allowed to incubate for 120 and 240 min at 37 °C. After the incubation, cells were washed with ice-cold PBS. One acid wash (0.2 M acetic acid, 0.5 M NaCl, pH 2.6) was performed and collected to measure the membrane-bound fraction followed by a wash with ice-cold PBS. The cells were lysed using 1 M NaOH and collected to measure the internalized fraction. These fractions were counted for activity using a Hidex gamma counter. The cells in the untreated plate were trypsinized and counted to determine the average number of cells per well. All experiments were performed twice in triplicates.

IC_50_ displacement assays were performed on LNCaP cells which were incubated with [^165^Er]Er-PSMA-617 (6 kBq, 0.2 nM) together with increasing concentrations (10^−13^ M–10^−6^ M) of unlabeled PSMA-617 in triplicate in a solution of 20 mM HEPES and 2 mg/mL bovine serum albumin in PBS (0.4 mL). The cells were incubated with activity for 3 h at 37 °C under CO_2_. After incubation, the supernatant was removed and the cells were washed with PBS. The cells were lysed with 1 M NaOH and the activity in each fraction was measured using a gamma counter. The data was plotted using Graphpad Prism Dose Response Inhibition plot. These experiments were performed twice in triplicates.

### ^165^Er SPECT/CT phantom imaging studies

The VECTor small animal imaging system is equipped with three large-field-of-view NaI gamma detectors in a triangular arrangement and X-ray CT. Cylindrical collimators with rings of clustered pinholes can be interchanged and optimized for resolution and sensitivity to best suit different energies from gamma emissions or annihilation photons in various preclinical applications (Goorden et al. 2012, Beekman and Vastevhouw 2002).

In this work, the feasibility of in vivo quantitative ^165^Er SPECT imaging was investigated utilizing the extra ultra-high sensitivity (XUHS) collimator. The XUHS collimator was proposed for pre-clinical ^165^Er SPECT since it is suitable for low energies (< 350 keV) and provides very high sensitivity, favourable for low-activity imaging (Ivashchenko et al. 2015). The XUHS collimator is a lead cylinder with an inner diameter of 46 mm and consists of 54 conical pinholes with a 2.0 mm diameter positioned in four rings. The central field of view (CFOV) size is 12 × 12 × 7 mm^3^.

The VECTor system acquires raw detector count data in list-mode format and allows the user to generate projection images with different energy-window settings. The ^165^Er photopeak projection images were generated from all its X-ray emissions between 46 and 56 keV (Table 1, total intensity ~ 74%) as the energy resolution of the system was not sufficient to resolve these individual emissions in the detected energy spectrum (Bolcaen et al. 2023). The photopeak window was centered at 47 keV and its width was set to 20%. Additionally, 2.5% wide lower and upper scatter windows were set for scatter and background correction using the triple energy window method.

**Table 1:** All x-rays emitted by ^165^Er and their intensities

Radionuclide	X-ray energy (keV)	Intensity (%)
^165^Er	6.7	17.0
	46.7	21.5
	47.5	37.9
	53.7	3.99
	53.9	7.73
	55.3	2.59

A pixel-based ordered subset expectation maximization (POSEM) iterative reconstruction algorithm (16 subsets, 6 iterations) was used to reconstruct the SPECT images with 0.4 mm^3^ voxel size. All animal images were reconstructed using these parameters (Branderhorst et al. 2010).

SPECT images were corrected for background and scatter by the triple energy window method (Koniar et al. 2022) and for attenuation by leveraging a CT-derived attenuation map generated on the scanner. Reconstructed images were analyzed with AMIDE software version 1.0.6. To generate quantitative ^165^Er SPECT images, the system was calibrated utilizing a 193.3 MBq/mL (10.4 MBq in 0.0536 mL) ^165^Er point-source in an Eppendorf tube. The point-source was scanned for 10 min in a single bed position and its projection data was generated and reconstructed using the same corrections and parameters as those described above. Volumes of interest (VOI) were drawn in the SPECT images of the point source to correlate the average voxel intensity with the activity concentration determined by HPGe gamma spectroscopy. The calibration factor was subsequently applied to future SPECT images to convert reconstructed count rates into quantitative units of activity concentration (MBq/mL).

After calibration of the VECTor, uniformity and resolution phantoms were also imaged (Table 2).

**Table 2:** Parameters and purposes for each of the phantom imaging experiments

Phantom	Total activity (MBq)	Volume (mL)	Real activity concentration (MBq/mL)	Scan time (min)	Purpose
Point source	10.4	0.0536	193.3	10	Calibration
Uniformity	29.3	6.3035	4.6	60	Background variability and quantitative accuracy of large regions
Resolution	11.7	0.3334	35.0	35	Spatial resolution, contrast, and quantitative accuracy of small regions

#### Uniformity phantom

The quantitative accuracy and response uniformity of the reconstructed SPECT images was analyzed using a 10 mL syringe filled (15 mm in diameter) with 6.3 mL of 29.3 MBq ^165^Er activity (4.6 MBq/mL). A total of 7 thin and long cylindrical VOIs (diameter: 4 mm and length: 20 mm) and 7 short and fat cylindrical VOIs (diameter: 12 mm and length: 2.85 mm) were drawn in the syringe (Fig. 3B) and the mean voxel intensity was calculated from each VOI. The quantitative accuracy was calculated for each VOI using Eq. 3 (Koniar et al. 2022):

3 $$Accuracy\; \left( \% \right) = 100 - \left( {\left| {1 - \frac{{Calculated\;Activity\;Concentration \;\left( {\frac{MBq}{{mL}}} \right) }}{{Real\;Activity\;Concentration\; \left( {\frac{MBq}{{mL}}} \right) }}} \right|} \right) \times 100$$

The Eq. 3 used to calculate quantitative accuracy of ^165^Er in a uniformity phantom.

Image uniformity was assessed in terms of the coefficient of variation (calculated as the standard deviation divided by the mean) of the average voxel intensity in the 7 cylindrical VOIs described above. Additionally, a line profile through the center of the phantom image was obtained and compared against the true profile.

#### Resolution phantom

A hot rod phantom with clusters of cylindrical holes with diameters 1.70 mm, 1.50 mm, 1.30 mm, 1.10 mm, 0.95 mm, and 0.85 mm was used to assess the imaging system’s ability to resolve small objects with ^165^Er. All the rods were filled with 35.0 MBq/mL of ^165^Er (Table 2).

Cylindrical VOIs (length: 6 mm) were placed on and between the rods, with diameters 80% the physical diameter of hot rods (1.36 mm, 1.20 mm, 1.04 mm, 0.88 mm, 0.76 mm, and 0.68 mm) to analyze the resulting SPECT images and quantify the resolvability of small hot regions using the inter-rod contrast (C_d_) metric (Koniar et al. 2022):

4 $$Inter - Rod\;Contrast\;(C_{d} ) = \frac{{\bar{h}_{d} - \bar{c}_{d} }}{{\bar{h}_{d} }}$$

The Eq. 4 used to calculate inter-rod contrast in resolution phantoms.

In Eq. 4, d refers to the rod diameter, ${\overline{h}}_{d}$ is the VOI mean (MBq/mL) for all cylinders with diameter index d placed on top of hot rods, and ${\overline{c}}_{d}$ is the VOI mean (MBq/mL) for all cylinders placed in between the hot rods (cold areas). The inter-rod contrast was calculated for each cluster of rods.

The imaging system’s accuracy in quantifying activity concentrations in small regions was assessed using the recovery coefficient (RC). The RC can be calculated using Eq. 5 (Koniar et al. 2022):

5 $$Recovery\;Coefficient\;(RC) = \frac{{\bar{h}_{d} }}{{Real\;Activity\;Concentration\;\left( {\frac{{MBq}}{{mL}}} \right)}}$$

The Eq. 5 used to calculate inter-rod contrast in resolution phantoms.

To assess the effect of lower acquired total counts, either from lower activity concentrations or shorter scan times, images of the resolution phantom were reconstructed using 20%, 5%, and 1% of the total counts, simulating data acquired with activity concentrations of 7.0, 1.8, and 0.4 MBq/mL. Inter-rod contrast and recovery coefficient were also assessed in these images.

### ^165^Er in vivo SPECT/CT imaging studies

#### Tumor inoculation

Male NRG mice (21 weeks old, breed in house) were inoculated at BC Cancer Research institute with LNCaP prostate cancer cells (10 × 10^6^ cells per mouse) on the left shoulder and waited for tumors to grow to reach 8–10 mm (< 0.5 g) in diameter for subsequent experiments. Mice were transferred to Center for Comprehensive Medicine at the University of British Columbia eight days before the experiments. Animal studies were performed in accordance with the Canadian Council on Animal Care (CCAC) using the protocol approved by the Animal Care Committee (ACC) of the University of British Columbia (A20-0113 and A20-0132).

#### [^165^Er]ErCl_3_ preparation

[^165^Er]ErCl_3_ was prepared by the addition of ^165^Er (118.2 MBq, 350 µL) to a solution containing ammonium acetate (2 M, pH 5.50, 40µL) and NaOH (1 M, 17.5 µL) and diluted with injectable saline to give suitable doses (~ 150 MBq/mL) for preclinical studies. Quality control on the final [^165^Er]ErCl_3_ preparation were performed using radio-TLC, radio-HPLC, and HPGe gamma spectroscopy. The pH for all injected [^165^Er]ErCl_3_ solutions was between 6.0 and 6.5 as confirmed by pH strips and [^165^Er]ErCl_3_ was injected in chloride form.

#### [^165^Er]Er-PSMA-617 radioligand preparation

All sample preparations were performed using low-retention pipette tips and low protein binding Eppendorf tubes to minimize losses of the radioligand through surface adsorption. [^165^Er]Er-PSMA-617 was prepared by the addition of ^165^Er (118.2 MBq, 350 µL) to a solution containing ammonium acetate (2 M, pH 5.50, 40µL) and NaOH (1 M, 17.5 µL). PSMA-617 (10^–4^ M, 10 µL, 1 nmol) was added (5.0 to 5.5 final pH), and the reaction mixture was mixed at 90 °C for 15 min. Reaction completion was confirmed using radio-TLC (> 95%). To minimize the presence of any [^165^Er]ErCl_3_ and other impurities, the solution of radiolabeled [^165^Er]Er-PSMA-617 (417.5 µL) was loaded onto a Sep-Pak C18 Plus Light Cartridge (pre-conditioned with ethanol (10 mL), saline (10 mL)). The reaction vessel was rinsed with saline (1 mL) and loaded onto the Sep-Pak cartridge to ensure quantitative transfer. The Sep-Pak cartridge was washed with saline (10 mL), and the purified product was eluted with ethanol (150 µL). Purified [^165^Er]Er-PSMA-617 (112.2 MBq) was concentrated to ~ 50 µL under an N_2_ gas stream and diluted with injectable saline to give suitable doses (~ 150 MBq/mL) for preclinical studies. Quality control on the final [^165^Er]Er-PSMA-617 product was performed using radio-TLC (100% RCY), radio-HPLC (> 99% RCP), and HPGe gamma spectroscopy. The molar activity was calculated using Eq. 1 assuming percentage of PSMA-617 recovery after Sep-Pak purification is directly correlated to the percentage of [^165^Er]Er-PSMA-617 recovery.

#### In vivo SPECT/CT imaging and ex vivo biodistribution

Preclinical studies were conducted with male NRG mice bearing LNCaP tumors to evaluate the imageability of ^165^Er in vivo. For imaging studies, experiments were performed using four replicates (n = 4) for [^165^Er]Er-PSMA-617 and two mice (n = 2) for [^165^Er]ErCl_3_. Mice were immobilized using a tailveiner restrainer (Braintree Scientific Inc., Braintree, MA, USA) and administered with [^165^Er]Er-PSMA-617 (mouse 1: 14.3 MBq, 0.16 nmol, 100 µL-mouse 2: 20.8 MBq, 0.25 nmol, 150 µL-mouse 3: 20.4 MBq, 0.25 nmol, 150 µL-mouse 4 (biodistribution only): 13.9 MBq, 0.16 nmol, 100 µL) or [^165^Er]ErCl_3_ (mouse 1: 11.7 MBq, 100 µL-mouse 2 (biodistribution only): 12.7 MBq, 102 µL) via intravenous injection to the lateral tail vein. Mice were anesthetized and maintained under a continuous stream of isoflurane (1.5–2.0% in oxygen) throughout the acquisition of the SPECT/CT scans, and their body temperature was kept using a heated animal bed. Quantitative static SPECT/CT scans were acquired immediately (15 min scan), 2.5 h (20 min scan), and 5 h (25 min scan) after the injection using a VECTor/CT multimodal preclinical scanner with an XUHS collimator. The mice were allowed to roam freely in their cages in between scans. SPECT data were reconstructed as described in Sect. "165Er SPECT/CT phantom imaging studies". Each imaging scan was decay corrected to the time of injection. After the acquisition of the final SPECT/CT scans, mice were kept under isoflurane and euthanized by CO_2_ asphyxiation, followed by cardiac puncture to recover blood activity, and relevant organs were harvested for biodistribution studies. From the SPECT/CT scans, mean standardized uptake values (SUV_mean_) were extracted for VOIs in the tumor, kidneys, and bladder. SUV_mean_ is calculated using Eq. 6:

6 $$\begin{aligned} SUV_{{mean}} \;\left( {\frac{g}{{mL}}} \right) = & \frac{{Radio\;activity\;Concentration\;\left( {\frac{{MBq}}{{mL}}} \right)\;in\;VOI}}{{Injected\;Activity\;\left( {MBq} \right)}} \\ & \times Animal\;Weight\;\left( g \right) \\ \end{aligned}$$

The Eq. 6 used for mean SUV calculation.

The activity concentration is calculated by multiplying VOI voxel intensities obtained from AMIDE software with the calibration factor of the instrument obtained for ^165^Er using the point source. The %IA/mL (injected activity/mL body tissue) in relevant organs was calculated directly from the quantitative SPECT/CT scans using Eq. 7:

7 $$\% IA/mL = \frac{{Radio\;activity\;Concentration\;\left( {\frac{{MBq}}{{mL}}} \right)in\;VOI}}{{Injected\;Activity\;\left( {MBq} \right)}} \times 100$$

The Eq. 7 used for %IA/mL calculations.

The %IA/g (injected activity/gram body tissue) in relevant organs was calculated directly from the quantitative SPECT/CT scans using Eq. 8:

8 $$\%IA/g=\frac{\%IA/mL}{Organ Density (\frac{g}{mL})}$$

The Eq. 8 used for %IA/g calculations.

The organ densities for NRG mice were obtained from literature (Davies and Morris 1993 and Foster et al. 1983). Maximum intensity projections (MIPs) for each time point were generated using AMIDE software, where a Gaussian filtering of full width half maximum (FWHM) = 1 mm was used for image rendering.

## Results

### ^165^Er production, dissolution, and purification

^165^Er was produced via the proton bombardment of ^165^Ho targets using a 13 MeV cyclotron at TRIUMF, with a production yield of 25 ± 5 MBq. µA^−1^.h^−1^ at EoB. ^165^Er was purified using a sequential combination of cation exchange (analytical grade 50W-X8) and extraction chromatography (TK212, TK211, and TK221). The purification process was slightly modified from the published method (Saeedi Saghez et al. 2024). Firstly, instead of collecting 250 mL of α-HIB from cation exchange resin for further purification, only 200 mL was collected. The last 50 mL fraction was determined to have low amounts of ^165^Er and was not collected to save time. In addition, the pH of α-HIB used for cation exchange elution was restricted to 4.70–4.75, which helped with more consistent ^165^Er recovery. This purification process is 4.0 ± 0.5 h long and has a decay corrected (EoB) ^165^Er recovery of 80.2 ± 4.5%. The final ^165^Er product does not contain other gamma-emitting radionuclides. The main stable impurities are natural Er and Ho (Saeedi Saghez et al. 2024).

### ^165^Er activity escalation and radiolabeling with PSMA-617

The radiolabeling tests for PSMA-617 with increasing activities of ^165^Er were performed to determine the highest molar activity achievable (Table 3).

**Table 3:** The results of labeling PSMA-617 with escalating activities of ^165^Er

Test ID	PSMA-617 mol (nmol)	Activity used (MBq)	Radiochemical yield (%)	Molar activity at EoS (MBq/nmol PSMA-617)	Molar activity corrected to EoB (MBq/nmol PSMA-617)
Test 1	0.10	3.9	100 ± 0	39.4 ± 0.0	67.3 ± 0.0
Test 2	0.10	8.4	100 ± 0	83.7 ± 0.0	143.0 ± 0.0
Test 3	0.10	12.8	98.3 ± 2.4	125.9 ± 3.1	215.0 ± 5.3
Test 4	0.10	17.2	98.7 ± 0.2	170.1 ± 0.4	290.6 ± 0.7
Test 5	0.10	21.7	94.2 ± 6.3	204.2 ± 13.6	348.8 ± 23.3
Test 6	0.10	26.1	67.6 ± 3.7	176.5 ± 9.6	301.4 ± 16.3
Test 7	0.10	44.3	45.1 ± 5.4	200.0 ± 24.0	341.6 ± 41.1

This experiment showed a maximum molar activity of 204.2 ± 13.6 MBq per nmol of PSMA-617 at the end of synthesis can be achieved (348.8 ± 23.3 MBq/nmol at the end of bombardment (EoB)).

### [^165^Er]Er-PSMA-617 distribution coefficient

The average distribution coefficient (LogD_7.4_) value for [^165^Er]Er-PSMA-617 was measured at −2.34 ± 0.24 (Table 2S in Supporting Information), suggesting that this radioligand is highly hydrophilic. Log*D*_7.4_ result is necessary to predict the excretion pathway of the radioligand in vivo. Hydrophilic compounds excrete through the kidneys and bladder pathway, which is expected from PSMA-617 radioligands.

### [^165^Er]Er-PSMA-617 stability in saline and serum

The kinetic stability tests with saline, human serum, and mice serum were performed with radio-TLC and radio-HPLC (all performed in triplicates).

**Fig. 2:**
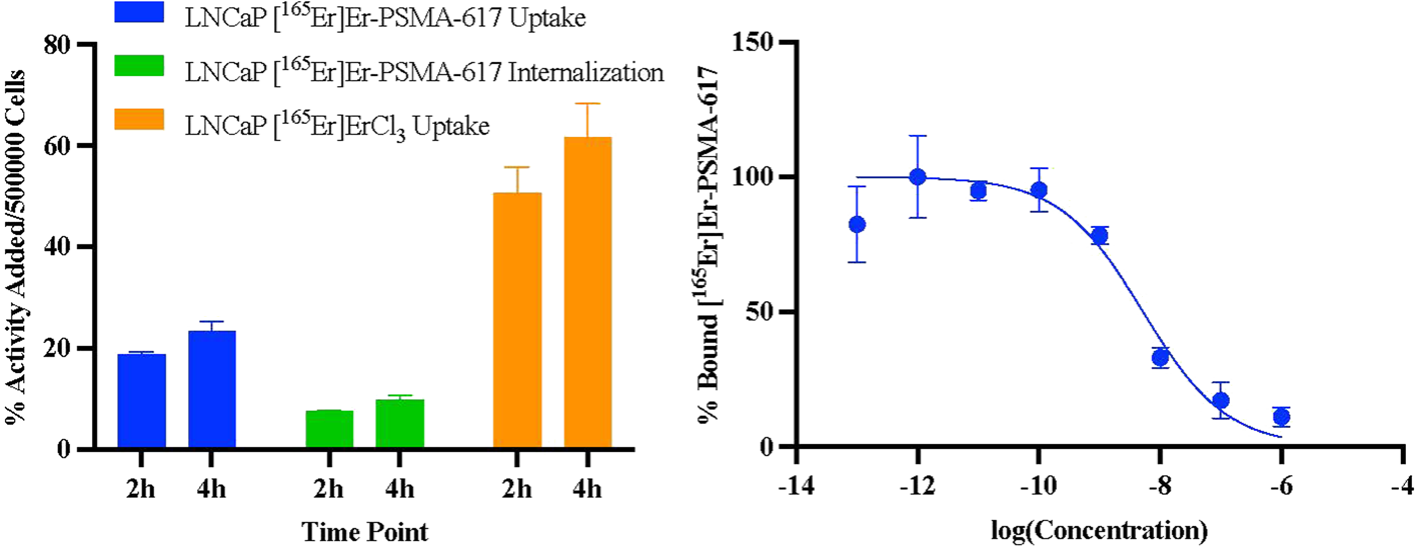
The uptake and internalization of [^165^Er]Er-PSMA-617 and uptake of [^165^Er]ErCl_3_ in LNCaP cells (left) and the plot of bound [^165^Er]Er-PSMA-617 as a function of increasing PSMA-617 concentration in LNCaP cells (right). All experiments performed twice in triplicates

[^165^Er]ErCl_3_ has a retention time of 1.6 min (solvent front) on the gamma trace and [^165^Er]Er-PSMA-617 has a retention time of 8.1 min under the same conditions (Fig. 1, individual HPLC tracers in Fig. 2S–4S in Supporting Information). [^nat^Er]Er-PSMA-617 retention time is of 7.9 min (ultra violet (UV) 220 nm). The discrepancy is consistent with the gamma detector located after the UV detector with a delay of 0.2 min at 1 mL/min.

The radio-HPLC gamma traces were obtained for [^165^Er]Er-PSMA-617 incubated with (× 10 v/v) saline, mice serum, and human serum. As the radio-HPLC traces showed, [^165^Er]Er-PSMA-617 remained a single peak with a retention time of about 8.1 min under all three conditions. Radio-TLC results also confirmed that ~ 100% of ^165^Er remained labeled after 12 h of incubation in all stability reactions.

### In vitro uptake, internalization, and IC_50_ displacement assays in LNCaP cells

The uptake of the radioligand needed to be confirmed by cell uptake assays before moving to animal studies. [^165^Er]Er-PSMA-617 showed uptake at 23.3 ± 2.1% activity per 5 × 10^5^ cells at 4 h in LNCaP cells which have a high density of PSMA receptors, and 9.7 ± 1.0% activity per 5 × 10^5^ cells internalized after 4 h of incubation (Fig. 2, left).

Interestingly, [^165^Er]ErCl_3_ has high uptake in LNCaP cells. Since [^165^Er]ErCl_3_ can be taken up by the cells regardless of receptor densities and can change the results of biodistribution and SPECT imaging. Therefore, purification of radioligand is required to separate residual unlabeled [^165^Er]ErCl_3_ from labeled [^165^Er]Er-PSMA-617.

In order to investigate if [^165^Er]Er-PSMA-617 uptake can be blocked with PSMA-617 and assess the relative binding affinity, the IC_50_ of PSMA-617 for [^165^Er]Er-PSMA-617 was measured. Increasing concentrations of PSMA-617 block the PSMA receptors on LNCaP cells and reduces the percentage of [^165^Er]Er-PSMA-617 bound to LNCaP cells (Fig. 2, right). Based on the data, the IC_50_ of PSMA-617 for [^165^Er]Er-PSMA-617 was calculated to be between 3.4 and 8.1 nM (95% confidence interval) and the best fit showing an IC_50_ value of 5.2 nM.

### ^165^Er SPECT/CT phantom imaging studies

The SPECT imaging properties of ^165^Er in a preclinical setting, including the uniformity, quantitation accuracy, and resolution, were assessed using small phantoms. All SPECT images were calibrated with a point source (Sect. "165Er SPECT/CT phantom imaging studies") and reconstructed using the 47 keV photopeak seen in the energy spectrum of ^165^Er (Supporting Information Fig. 5S).

#### Uniformity phantom

**Fig. 3:**
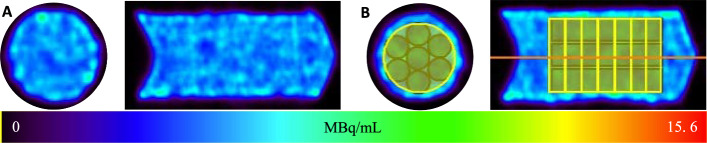
The SPECT images acquired for ^165^Er placed in a syringe with a concentration of 4.6 MBq/mL at the time of acquisition. A) top and side views. B) cylindrical VOIs (dark yellow) drawn for quantitative accuracy and noise calculations and cylindrical VOIs (light yellow) drawn for background calculation. The fly through line used to generate the uniformity profile graph shown on the side view

**Fig. 4:**
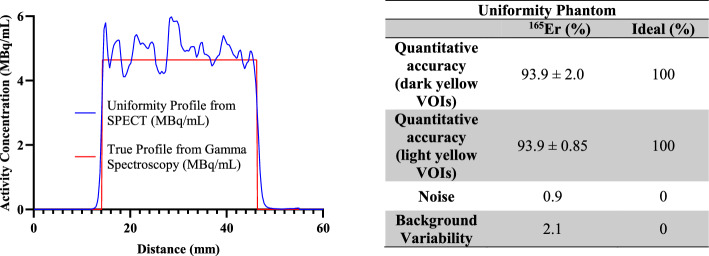
Left: activity concentration profile through the uniformity region (blue) and the true activity concentration (red). Right: quantitative results obtained from uniformity phantom of ^165^Er

The uniformity phantom was a 10 mL syringe filled with 6 mL solution of ^165^Er (Fig. 3). The average quantitative accuracy for calculated activity concentrations of dark yellow (long and thin) and bright yellow (short and fat) VOIs were 93.9 ± 2.0% and 93.9 ± 0.85% respectively, showing good calibration and high accuracy for quantitative SPECT imaging with ^165^Er. The noise was calculated to be 0.9% and the background variability was calculated to be 2.1% using coefficient of variation metric. The uniformity profile (activity concentration across the middle of the phantom) from SPECT is close to the true profile obtained from gamma spectroscopy (Fig. 4).

**Table:** 

Uniformity phantom		
	^165^Er (%)	Ideal (%)
Quantitative accuracy (dark yellow VOIs)	93.9 ± 2.0	100
Quantitative accuracy (light yellow VOIs)	93.9 ± 0.85	100
Noise	0.9	0
Background variability	2.1	0

#### Resolution phantom

**Fig. 5:**
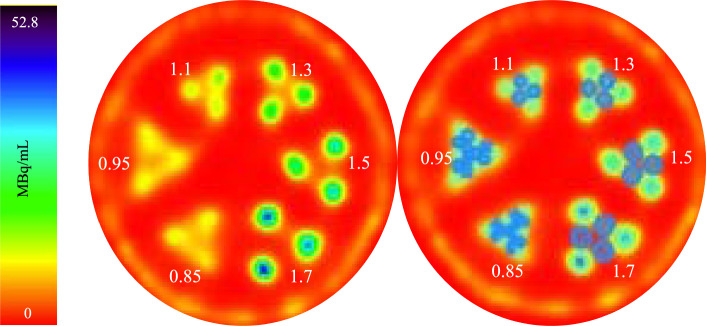
The SPECT image of a ^165^Er resolution phantom obtained and VOI placements on top of hot rods (green) and in between rods (blue) for inter-rod contrast, recovery coefficient, and noise measurements

The resolution phantom has six clusters of cylindrical holes filled with activity (35.0 MBq/mL) (Fig. 5). The diameters of the holes are 1.70 mm, 1.50 mm, 1.30 mm, 1.10 mm, 0.95 mm, and 0.85 mm. VOIs were drawn on top of the hot rods and in between of the hot rods. The average activity concentration across hot rods of a particular diameter was calculated. This was also done for VOIs in between hot rods. Based on calculated mean activity concentration and the real activity concentration (MBq/mL), the recovery coefficient and inter-rod contrast were calculated (Table 4).

**Table 4:** The inter-rod contrast and recovery coefficient calculations for ^165^Er resolution phantom

Phantom	Rod diameter (mm)	Calculated mean activity concentration in hot VOIs (MBq/mL)	Calculated mean activity concentration in cold VOIs (MBq/mL)	Inter-rod contrast (C_d_)	Recovery coefficient (RC)
Resolution	1.7	28.6	3.2	0.89	0.82
	1.5	23.9	4.8	0.80	0.68
	1.3	18.3	6.7	0.64	0.52
	1.1	12.3	8.6	0.30	0.35
	0.95	9.6	9.2	0.04	0.27
	0.85	9.5	9.4	− 0.01	0.27

The ideal value for both inter-rod contrast and recovery coefficient is 1. However, any rod with C_d_ values above 0.2 would be visually resolved (Koniar et al. 2022). This can also be seen in the SPECT image (Fig. 5, left), where rods with diameters as low as 1.1 mm are visually resolved but rods with diameters of 0.95 mm and 0.85 mm are not resolved. The calculations further confirm this observation as rods with diameter of 1.1 mm have inter-rod contrast of 0.3 making them resolvable but smaller rods have much lower C_d_ and are not resolvable.

**Fig. 6:**
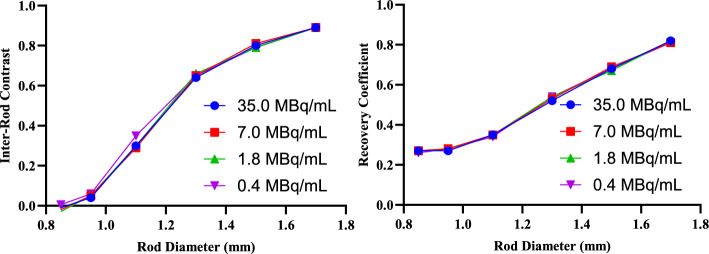
The inter-rod contrast and recovery coefficient as a function of hot rod diameters reconstructed at different activity concentrations

Reconstruction of resolution phantom at lower number of counts showed no significant difference in inter-rod contrast and recovery coefficient values from 0.4 MBq/mL to 35.0 MBq/mL, indicating that ^165^Er maintains this resolution at this concentration range for a 35-min SPECT acquisition (Fig. 6).

### ^165^Er in vivo SPECT/CT imaging studies

#### In vivo ^165^Er SPECT/CT imaging and ex vivo biodistribution

**Fig. 7:**
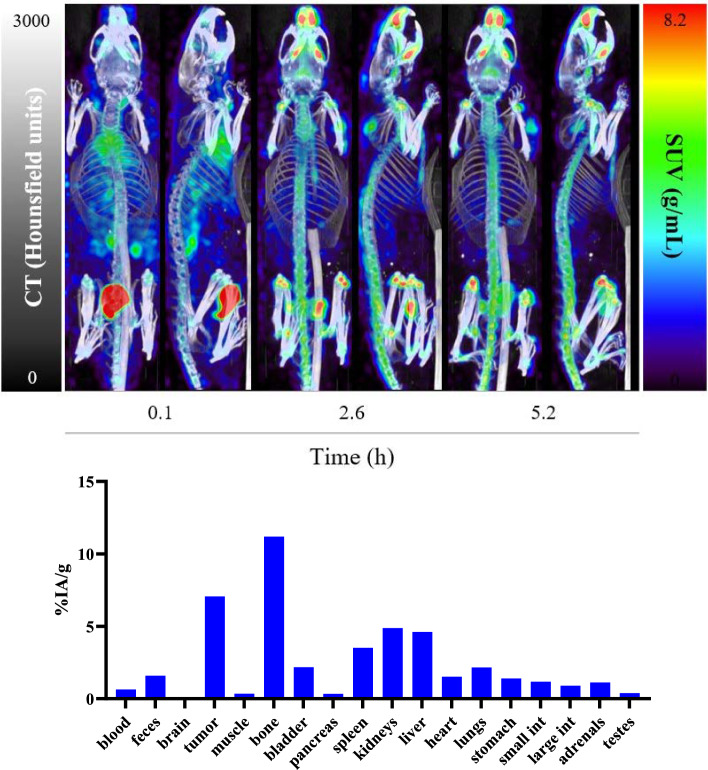
MIP-sagittal and coronal views from quantitative static SPECT/CT scans recorded at 0.1, 2.6, and 5.2 h post-injection of [.^165^Er]ErCl_3_ (11.7 MBq) and biodistribution in LNCaP tumor-bearing mice at 5.2 h post-injection (n = 2)

To discover if ^165^Er is imageable in vivo, and also determine the distribution of Er^3+^ in mice, SPECT/CT studies were performed with [^165^Er]ErCl_3_ in an LNCaP tumor-bearing mouse. The SPECT image (MIP) for this mouse was acquired and the ex vivo biodistribution in all major organs at 5.2 h post-injection are examined (Fig. 7, Supporting Information Table 3S).

The SPECT/CT images show that bone and spine have a significant accumulation of [^165^Er]ErCl_3_. It was also noted that there was considerable liver and spleen uptake at 5.2 h. As a free ion, Er^3+^ can be bound by serum proteins and travel to different organs, which explains the uptake by multiple organs. The uptake of [^165^Er]ErCl_3_ in the tumor was also notable, which is not surprising considering the in vitro experiments that showed significant activity uptake in LNCaP cells and the fact that the tumor is a highly vascular organ.

When comparing the SPECT calculations with ex vivo biodistribution results, the quantification obtained from the SPECT/CT scans showed good consistency with the ex vivo biodistribution measurements (Table 5).

**Table 5:** Comparison of the activity concentrations in VOIs measured via quantitative SPECT/CT vs. ex vivo biodistribution studies at 5.2 h post-injection for [^165^Er]ErCl_3_

Organ	Density (g/mL)	SPECT/CT			BioD
		SUV (g/mL)	%IA/mL	%IA/g	%IA/g
Tumor	1	3.89	8.73	8.73	7.42
Kidneys	1.06	1.03	3.23	3.07	4.01
Bladder + Urine	1.03	5.74	18.1	17.5	15.0
Liver	1.05	1.52	4.79	4.52	4.46
Bone	1.92	104	24.4	12.7	9.89
Spleen	1	1.78	5.59	5.59	5.23

#### In vivo [^165^Er]Er-PSMA-617 SPECT/CT imaging and ex vivo biodistribution

**Fig. 8:**
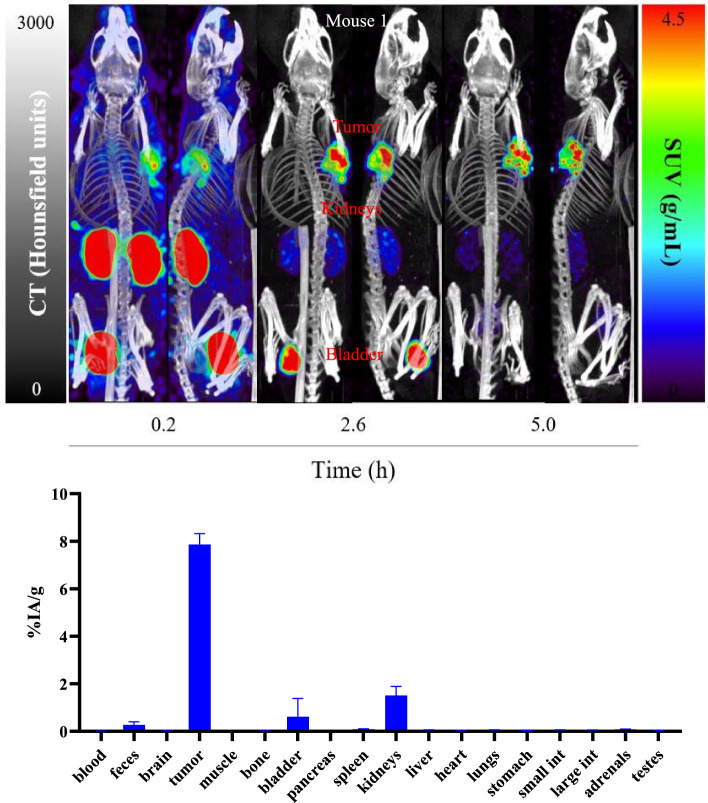
MIP-sagittal and coronal views from quantitative static SPECT/CT scans recorded at 0.2, 2.6, and 5.0 h post-administration of [.^165^Er]Er-PSMA-617 (20.4 MBq, 0.25 nmol, n = 3) and biodistribution results in LNCaP tumor-bearing mice at 5.2 h (n = 4)

[^165^Er]Er-PSMA-617 was administered to four LNCaP tumor-bearing mice (left-shoulder), and a series of static quantitative SPECT/CT scans were acquired 0.1, 2.5, and 5 h after the injection (Figs. 8 and 6S in Supporting Information), and the activity concentrations in tumor, kidneys and bladder + urine were calculated for the three mice (Table 6). The ex vivo biodistribution (n = 4) in organs and tissues at 5.0 h post-injection was studied for comparison (Fig. 8, Tables 6 and 3S in Supplementing Information).

**Table 6:** The comparison of %IA/g from SPECT/CT with ex vivo biodistribution for each mouse at 5 h post-injection of [^165^Er]Er-PSMA-617

Mice #	Organ	Density (g/mL)	SPECT			BioD	Quantitative accuracy (%)
			SUV (g/mL)	%IA/mL	%IA/g	%IA/g	
Mouse 1	Tumor	1	2.82	9.13	9.13	8.13	87.7
	Kidneys	1.06	0.28	0.92	0.88	1.22	72.1
	Bladder + Urine	1.03	0.49	1.57	1.53	2.50	61.2
Mouse 2	Tumor	1	2.64	9.14	9.14	7.48	77.8
	Kidneys	1.06	0.36	1.24	1.18	2.03	58.1
	Bladder + Urine	1.03	0.54	1.85	1.80	2.70	66.7
Mouse 3	Tumor	1	3.18	9.51	9.51	8.36	86.2
	Kidneys	1.06	0.47	1.41	1.34	1.23	91.1
	Bladder + Urine	1.03	17.4	52.0	50.5	46.7	91.9

The SPECT/CT images showed rapid uptake of [^165^Er]Er-PSMA-617 in LNCaP tumors at 5–10 min post-administration (SUV_mean_ = 2.00 ± 0.16 g/mL), which increased over time reaching a maximum at 2.5 h post-injection (SUV_mean_ = 2.83 ± 0.27 g/mL at 2.5 h and SUV_mean_ = 2.88 ± 0.27 g/mL at 5 h). Quantification of the SPECT/CT scans revealed a tumor uptake of 9.26 ± 0.22%IA/g (n = 3) at 5.0 h post-injection, which was consistent with the ex vivo biodistribution measurements (8.36 ± 0.57%IA/g, n = 4). Further quantification of images for kidneys and bladder at 5 h post-injection revealed an uptake of 1.13 ± 0.23%IA/g and 17.9 ± 28.2%IA/g (n = 3), respectively, which were in agreement with ex vivo measurements (1.79 ± 0.46%IA/g for kidneys and 14.7 ± 21.6%IA/g for bladder combined with urine (n = 4)). When examine the individual numbers, while the higher uptake regions showed good quantitative accuracy (77.8% to 91.9%), the lower uptake regions (< 2.5%IA/g) have lower quantitative accuracy (58.1% to 91.1%).

**Fig. 9:**
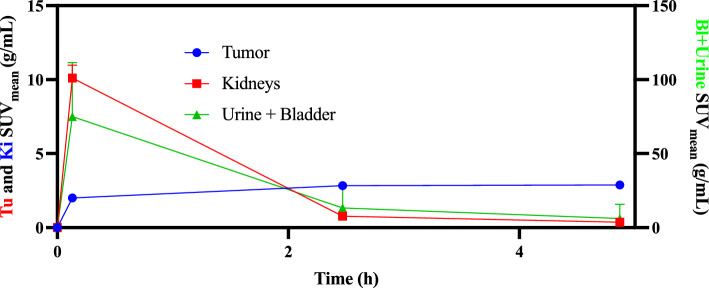
Time-activity curves for [^165^Er]Er-PSMA-617 in SUV_mean_ as a function of time in male NRG mice bearing LNCaP tumor. Tu: Tumor; Ki: Kidneys; Bl: Bladder

From the SPECT images over time for all three mice injected with [^165^Er]Er-PSMA-617, the mean activity concentrations in VOIs placed on tumor, kidneys, and urine + bladder were calculated (Table 7), and the SUV_mean_ values were plotted over time (Fig. 9).

**Table 7:** The mean activity concentrations in VOIs calculated as SUV_mean_ (g/mL) and %IA/g via quantitative SPECT/CT at different time points

Time (h)	Tumor SPECT		Kidneys SPECT		Bladder + Urine SPECT	
	SUVmean (g/mL)	%IA/g	SUVmean (g/mL)	%IA/g	SUVmean (g/mL)	%IA/g
0	0	0	0	0	0	0
0.13 ± 0.06	2.00 ± 0.16	6.43 ± 0.34	10.1 ± 0.87	31.1 ± 2.7	75.0 ± 36.5	240 ± 133
2.47 ± 0.12	2.83 ± 0.27	9.09 ± 0.27	0.77 ± 0.14	2.39 ± 0.56	13.3 ± 15.1	39.7 ± 43.1
4.87 ± 0.15	2.88 ± 0.27	9.26 ± 0.22	0.37 ± 0.09	1.13 ± 0.23	6.13 ± 9.73	17.9 ± 28.2

[^165^Er]Er-PSMA-617 showed rapid clearance from the bloodstream, with rapid elimination through the renal pathway, which was expected as the radioligand is highly hydrophilic. This is evidenced by a rapid decline of activity within the kidneys and bladder when comparing the 0.1 h and 2.5 h images and is supported by the time–activity curves (Fig. 9). Most of the radioligand was cleared from circulation and tumor uptake reached a plateau at 2.5 h post-injection.

## Discussion

Using the ^165^Er purified with the slightly modified method, PSMA-617 was labeled with molar activities up to 200 MBq/nmol. The hydrophilicity of [^165^Er]Er-PSMA-617 radioligand was determined with an average Log*D*_7.4_ value of -2.34 ± 0.24. This value is similar to the value reported by Uygur et al. 2023 for [^161^ Tb]Tb-PSMA-617 (−2.15 ± 0.13), but differs from that reported for [^165^Er]Er-PSMA-617 (−3.3 ± 0.3, Da Silva et al. 2021), [^161^ Tb]Tb-PSMA-617 (− 3.9 ± 0.1, Müller et al. 2019), and [^177^Lu]Lu-PSMA-617 (−4.18 ± 0.06, Silva et al. 2021). This discrepancy might be due to the low activity in the n-octanol layer for highly hydrophilic compounds or due to differerences in detection equipment (Uygur et al. 2023). Nonetheless, the produced radioligand is highly hydrophilic and renal clearance is expected.

The stability of [^165^Er]Er-PSMA-617 complex in saline, human serum, and mice serum was analyzed over one half-life of ^165^Er using both radio-TLC and radio-HPLC. Radio-TLC showed that ^165^Er remained bound to PSMA-617 without any presence of free activity. Radio-TLC does not provide information on the integrity of PSMA-617 (degraded or intact) and performing radio-HPLC is necessary to determine how stable the whole complex is over time. Radio-HPLC showed no evidence of PSMA-617 degradation, hydrolysis, or release of free activity. This result is consistent with the report [^165^Er]Er-PSMA-617 is stable in human serum over 12 h (Silva et al. 2021).

Preliminary in vitro cell uptake and internalization confirmed that the behavior of [^165^Er]Er-PSMA-617 (23.3 ± 2.1% activity per 5 × 10^5^ cells uptake and 9.7 ± 1.0% internalization at 4 h) is comparable to reported [^58^Co]Co-PSMA-617 (~ 50% activity per 10^6^ cells uptake and 27.9 ± 2.1% activity per 10^6^ cells internalization) and [^177^Lu]Lu-PSMA-617 (3–3.5% activity per 1 × 10^5^ cells uptake) (Baun et al. 2023; Ruigrok et al. 2020) in LNCaP cells. Furthermore, the IC_50_ values for [^165^Er]Er-PSMA-617 was in the nanomolar range which is comparable to [^177^Lu]Lu-PSMA-617 counterpart (Ruigrok et al. 2020). Interestingly, the in vitro uptake data with [^165^Er]ErCl_3_ showed high uptake in LNCaP cells as well.

Phantom imaging demonstrated ^165^Er as a preclinical SPECT imaging radionuclide. Reconstructed ^165^Er SPECT images have quantitative accuracy of 94%. Resolution phantom imaging showed that structures of 1.1 mm or larger could be differentiated in ^165^Er pre-clinical SPECT, similar to results reported for ^155^ Tb SPECT (Favaretto et al. 2021 and Koniar et al. 2024). A head-to-head comparison with ^177^Lu is yet to be investigated, and whether this can be translated to a clinical scanner is yet to be determined. Significant attenuation of X-ray emissions from ^165^Er is expected in patients, which may be countered by the high yield of these emissions (74% for ^165^Er compared to 10.4% of the 208 keV peak of ^177^Lu) and the enhanced detection efficiency of NaI detector crystals at lower energy levels.

Using the high abundance X-ray emissions from ^165^Er, the SPECT/CT in vivo images of [^165^Er]ErCl_3_ were acquired, demonstrating the applicability of ^165^Er for preclinical imaging. The SPECT imaging with [^165^Er]ErCl_3_ showed significant accumulations in skeleton and clearance occurs through both the liver and kidneys. Considering the reports that [^132/135^La]LaCl_3_ was taken up primarily in liver with low bone uptake (Aluicio-Sarduy 2019), [^161^Tb]TbCl_3_ had significant bone and liver uptake (Cassells 2021), and [^177^Lu]LuCl_3_ showed predominant bone uptake (Repetto-Llamazares, 2013), the results suggest the trend of increased bone uptake and decreased liver uptake throughout lanthanide series.

Finally, the SPECT/CT imaging with [^165^Er]ErCl_3_ and [^165^Er]Er-PSMA-617 demonstrated the use of ^165^Er for preclinical imaging applications along with its good imaging contrast, resolution, and quantitative accuracy. The [^165^Er]Er-PSMA-617 biodistribution profile was similar to previous reports for [^177^Lu]Lu-PSMA-617 in the same model (Kuo et al. 2018), showing good uptake in tumor and kidneys, and low uptake in other organs. The absolute values are different. Kuo et al. reports 14.5 ± 1.83%IA/g tumor uptake at 4 h post injection for [^177^Lu]Lu-PSMA-617 while we saw 7.84 ± 0.46%IA/g tumor uptake at 5 h post injection for [^165^Er]Er-PSMA-617. The discrepancy may be attributed to differences in molar activity of radioligands injected (780 MBq/nmol for [^177^Lu]Lu-PSMA-617, 100 MBq/nmol of [^165^Er]Er-PSMA-617).

Overall, ^165^Er could play an important role in preclinical SPECT/CT imaging considering the starting ^165^Ho material is inexpensive and cyclotron production yields are in GBq scale for short proton irradiations. Since ^165^Er is a pure Auger electron emitter, it holds promise as a true theranostic for preclinical targeted imaging and radionuclide therapy of cancer using the same radioligand.

## Conclusions

These studies showed small medical cyclotron-produced ^165^Er can be used for investigating radiochemistry, in vitro uptake, and in vivo distribution of a tumor targeting vector, including SPECT imaging. Further investigations, including the simulation study for potential clinical applications, are ongoing.

## Supplementary Information

Additional file 1.

## Data Availability

The datasets supporting the conclusions of this article are included within the article and its Supporting Information. Raw data are available upon reasonable request.

## Abbreviations

ACC: Animal care committee
CFOV: Central field of view
CPS: Counts per second
CT: Computed tomography
DI: Deionized
DOE: Department of energy
EDTA: Ethylenediaminetetraacetic acid
FBS: Fetal Bovine serum
FWHM: Full width half maximum
α-HIB: Alpha-hydroxyisobutyrate
HPGe: High purity germanium
HPLC: High-performance liquid chromatography
IA: Injected activity
MA: Molar activity
MIP: Maximum intensity projection
PBS: Phosphate buffered saline
PET: Positron emission tomography
POSEM: Pixel-based ordered subset expectation maximization
PS: Penicillin–streptomycin
PSMA: Prostate specific membrane antigen
RCY: Radiochemical yield
RCP: Radiochemical purity
VOI: Volume of interest
RPM: Rounds per minute
RPMI: Roswell park memorial institute
SA: Silicic acid
SPECT: Single photon emission computed tomography
SUV: Standardized uptake value
TFA: Trifluoroacetic acid
TLC: Thin layer chromatography
UV: Ultra violet
XUHS: Extra ultra high sensitivity

## References

[CR1] Aluicio-Sarduy E, Hernandez R, Olson AP, et al. Production and in vivo PET/CT imaging of the theranostic pair ^132/135^La. Sci Rep. 2019;9:10658. 10.1038/s41598-019-47137-0. 31337833 10.1038/s41598-019-47137-0 PMC6650468

[CR2] Baun C, Dam JH, Hildebrandt MG, Ewald JD, Kristensen BW, Gammelsrød VS, et al. Preclinical evaluation of [58mCo]co-dota-psma-617 for auger electron therapy of prostate cancer. Sci Rep. 2023. 10.1038/s41598-023-43429-8. 37914790 10.1038/s41598-023-43429-8 PMC10620164

[CR3] Beekman FJ, Vastenhouw B. Design and simulation of U-SPECT, an ultra-high resolution molecular imaging system. In: 2002 IEEE nuclear science symposium conference record. 2002;2:792–6. 10.1109/nssmic.2002.1239444

[CR4] Bolcaen J, Gizawy MA, Terry SYA, Paulo A, Cornelissen B, Korde A, et al. Marshalling the potential of Auger electron radiopharmaceutical therapy. J Nucl Med. 2023;64(9):1344–51. 10.2967/jnumed.122.265039. 37591544 10.2967/jnumed.122.265039 PMC10478825

[CR5] Branderhorst W, Vastenhouw B, Beekman FJ. Pixel-based subsets for rapid multi-pinhole SPECT reconstruction. Phys Med Biol. 2010;55(7):2023–34. 10.1088/0031-9155/55/7/015. 20299722 10.1088/0031-9155/55/7/015

[CR6] Cassells I, Ahenkorah S, Burgoyne AR, Van de Voorde M, Deroose CM, Cardinaels T, Bormans G, Ooms M, Cleeren F. Radiolabeling of human serum albumin with Terbium-161 using mild conditions and evaluation of in vivo stability. Front Med. 2021;8:675122. 10.3389/fmed.2021.675122. 10.3389/fmed.2021.675122 PMC8422959 34504849

[CR7] Da Silva I, Johnson TR, Mixdorf JC, Aluicio-Sarduy E, Barnhart TE, Nickles RJ, et al. A high separation factor for ^165^Er from Ho for targeted radionuclide therapy. Molecules. 2021;26(24):7513. 10.3390/molecules26247513. 34946596 10.3390/molecules26247513 PMC8707915

[CR8] Davies B, Morris T. Physiological parameters in laboratory animals and humans. Pharm Res. 1993;10(7):1093–5. 10.1023/a:1018943613122. 8378254 10.1023/a:1018943613122

[CR9] Favaretto C, Talip Z, Borgna F, Grundler PV, Dellepiane G, Sommerhalder A, et al. Cyclotron production and radiochemical purification of terbium-155 for SPECT imaging. EJNMMI Radiopharm Chem. 2021. 10.1186/s41181-021-00153-w. 34778932 10.1186/s41181-021-00153-w PMC8590989

[CR10] Foster HL, Small JD, Fox JG. Preface. In: The mouse in biomedical research. New York: Elsevier; 1983. p. xiii–xiv.

[CR11] Goorden MC, van der Have F, Kreuger R, Ramakers RM, Vastenhouw B, Burbach JP, et al. Vector: a preclinical imaging system for simultaneous submillimeter SPECT and PET. J Nucl Med. 2012;54(2):306–12. 10.2967/jnumed.112.109538. 23077113 10.2967/jnumed.112.109538

[CR12] Gracheva N, Carzaniga TS, Schibli R, Braccini S, van der Meulen NP. ^165^Er: a new candidate for auger electron therapy and its possible cyclotron production from natural holmium targets. Appl Radiat Isot. 2020;159:109079. 10.1016/j.apradiso.2020.109079. 32068146 10.1016/j.apradiso.2020.109079

[CR13] Ivashchenko O, van der Have F, Goorden MC, Ramakers RM, Beekman FJ. Ultra-high-sensitivity submillimeter mouse SPECT. J Nucl Med. 2015;56(3):470–5. 10.2967/jnumed.114.147140. 25678487 10.2967/jnumed.114.147140

[CR14] Koniar H, Rodríguez-Rodríguez C, Radchenko V, Yang H, Kunz P, Rahmim A, et al. SPECT imaging of ^226^Ac as a theranostic isotope for ^225^Ac radiopharmaceutical development. Phys Med Biol. 2022;67(18):185009. 10.1088/1361-6560/ac8b5f. 10.1088/1361-6560/ac8b5f 35985341

[CR15] Koniar H, McNeil S, Wharton L, Ingham A, Van de Voorde M, Ooms M, et al. Quantitative SPECT imaging of ^155^Tb and ^161^Tb for preclinical theranostic radiopharmaceutical development. EJNMMI Phys. 2024. 10.1186/s40658-024-00682-8. 39276263 10.1186/s40658-024-00682-8 PMC11401819

[CR16] Kuo H-T, Merkens H, Zhang Z, Uribe CF, Lau J, Zhang C, et al. Enhancing treatment efficacy of 177lu-PSMA-617 with the conjugation of an albumin-binding motif: preclinical dosimetry and endoradiotherapy studies. Mol Pharm. 2018;15(11):5183–91. 10.1021/acs.molpharmaceut.8b00720. 30251544 10.1021/acs.molpharmaceut.8b00720

[CR17] Müller C, Umbricht CA, Gracheva N, Tschan VJ, Pellegrini G, Bernhardt P, et al. Terbium-161 for PSMA-targeted radionuclide therapy of prostate cancer. Eur J Nucl Med Mol Imaging. 2019;46(9):1919–30. 10.1007/s00259-019-043450. 31134301 10.1007/s00259-019-04345-0 PMC6820371

[CR18] National Nuclear Data Center [Internet]. [cited 2024 Sept 10]. Available from: http://www.nndc.bnl.gov./

[CR19] Repetto-Llamazares AH, Larsen RH, Mollatt C, Lassmann M, Dahle J. Biodistribution and dosimetry of (177)Lu-tetulomab, a new radioimmunoconjugate for treatment of non-Hodgkin lymphoma. Curr Radiopharm. 2013;6(1):20–7. 10.2174/1874471011306010004. 23256748 10.2174/1874471011306010004 PMC3624777

[CR20] Ruigrok EA, van Vliet N, Dalm SU, de Blois E, van Gent DC, Haeck J, et al. Extensive preclinical evaluation of lutetium-177-labeled PSMA-specific tracers for prostate cancer radionuclide therapy. Eur J Nucl Med Mol Imaging. 2020;48(5):1339–50. 10.1007/s00259-020-05057-6. 33094433 10.1007/s00259-020-05057-6 PMC8113296

[CR21] Saeedi Saghez B, Yang H, Radchenko V. High separation factor, high molar activity, and inexpensive purification method for the production of pure ^165^Er. Inorg Chem. 2024;63(12):5330–40. 10.1021/acs.inorgchem.3c03166. 38324916 10.1021/acs.inorgchem.3c03166

[CR22] Uygur E, Sezgin C, Parlak Y, Karatay KB, Arikbasi B, Avcibasi U, et al. The radiolabeling of [^161^Tb]-PSMA-617 by a novel radiolabeling method and preclinical evaluation by in vitro/in vivo methods. 2023. 10.21203/rs.3.rs-3415703/v1

[CR23] Zhang C, Zhang Z, Lin K-S, Pan J, Dude I, Hundal-Jabal N, et al. Preclinical melanoma imaging with ^68^Ga-labeled α-melanocyte-stimulating hormone derivatives using PET. Theranostics. 2017;7(4):805–13. 10.7150/thno.17117. 28382155 10.7150/thno.17117 PMC5381245

