# Supplementary material for: First preclinical SPECT/CT imaging and biodistribution of [165Er]ErCl3 and [165Er]Er-PSMA-617
Source: EJNMMI Radiopharm Chem. 2024 Dec 18;9:90. doi: 10.1186/s41181-024-00312-9 (PMC11655927; doi:10.1186/s41181-024-00312-9)
Supplement: Supplementary file 1 — Additional file 1. [file 41181_2024_312_MOESM1_ESM.docx]

**Supporting Information**

**First Preclinical SPECT/CT Imaging and Biodistribution of [^165^Er]ErCl_3_ and [^165^Er]Er-PSMA-617**

**Behrad Saeedi Saghez^1,2^, Cristina Rodríguez-Rodríguez^3,4^, Pedro Luis Esquinas^5^, Helen Merkens^6,7^, François Bénard^6,7^, Valery Radchenko^1,2^, and Hua Yang^1,2,8*^**

^1^ Life Sciences Division, TRIUMF, 4004 Wesbrook Mall, Vancouver, BC V6T 2A3, Canada;

^2^ Department of Chemistry, University of British Columbia, 2036 Main Mall, Vancouver, BC V6T 1Z1, Canada

^3^ Department of Physics and Astronomy, University of British Columbia, 6224 Agricultrual Road,

Vancouver, BC V6T 1Z1, Canada

^4^ Faculty of Pharmaceutical Sciences, University of British Columbia, 2405 Wesbrook Mall, Vancouver, BC V6T 1Z3, Canada

^5^ Department of Integrative Oncology, BC Cancer Research Institute, 675 West 10^th^ Avenue, Vancouver, BC V5Z 1L3, Canada

^6^ Department of Molecular Oncology, BC Cancer Research Institute, 675 West 10^th^ Avenue, Vancouver, BC V5Z 1L3, Canada

^7^ Department of Radiology, University of British Columbia, 2775 Laurel Street, Vancouver, BC V5Z 1M9, Canada

^8^ Department of Chemistry, Simon Fraser University, 8888 University Drive, Burnaby, BC V5A 1S6, Canada

* Correspondence: Hua Yang, [hyang@triumf.ca](mailto:hyang@triumf.ca)

1. **Conditions Used for [^165^Er]Er-PSMA-617 Radiolabeling and Dose Escalation**

The reaction conditions for [^165^Er]Er-PSMA-617 radiolabeling and dose escalation are summarized in Table 1S.

**Table 1S. The reaction conditions used for dose escalation reactions with PSMA-617 using a high activity concentration of 493 MBq/mL at the end of synthesis (obtained using a four hours irradiation of a 200 mg Ho target with 30 µA current and 12.8 MeV energy).**

| ID | PSMA-617 Molarity (M) | Volume of PSMA-617 Used (µL) | Volume of Buffer Used (µL) | Volume of Er-165 Used (µL) | Volume of NaOH (µL) | Activity Used (MBq) | PSMA-617 Moles (nmol) |
| --- | --- | --- | --- | --- | --- | --- | --- |
| Test 1 | 0.0001 | 1.00 | 1.00 | 8.00 | 0.40 | 3.9 | 0.10 |
| Test 2 | 0.0001 | 1.00 | 2.00 | 17.00 | 0.85 | 8.4 | 0.10 |
| Test 3 | 0.0001 | 1.00 | 3.00 | 26.00 | 1.30 | 12.8 | 0.10 |
| Test 4 | 0.0001 | 1.00 | 4.00 | 35.00 | 1.75 | 17.2 | 0.10 |
| Test 5 | 0.0001 | 1.00 | 5.00 | 44.00 | 2.20 | 21.7 | 0.10 |
| Test 6 | 0.0001 | 1.00 | 6.00 | 53.00 | 2.65 | 26.1 | 0.10 |
| Test 7 | 0.0001 | 1.00 | 9.00 | 90.00 | 4.50 | 44.3 | 0.10 |

In all reactions (prepared in duplicates), the amount of PSMA-617 was kept constant (0.1 nmol) while the activity added was gradually increased. Figure 1S shows the sample radio-TLC results for unlabelled Er-165 ([^165^Er]ErCl_3_) and [^165^Er]Er-PSMA-617. The plates are 110 mm long salicylic acid impregnated iTLC papers.

[^165^Er]Er-PSMA-617

[^165^Er]ErCl_3_


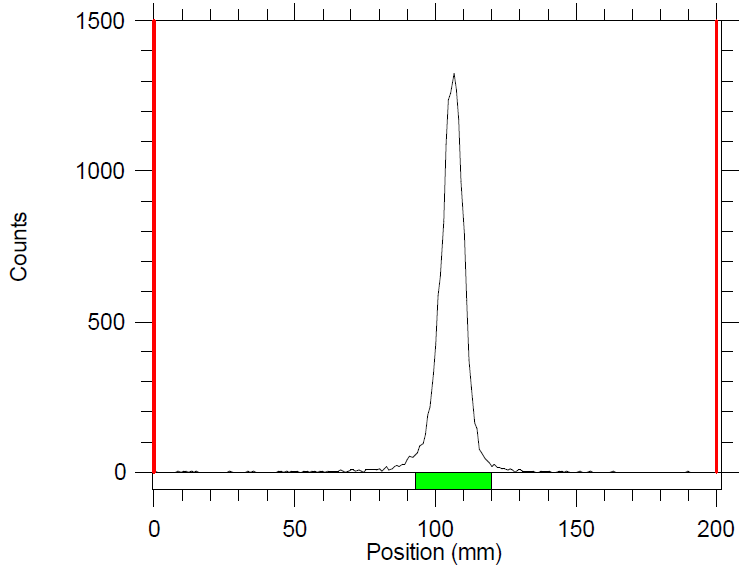

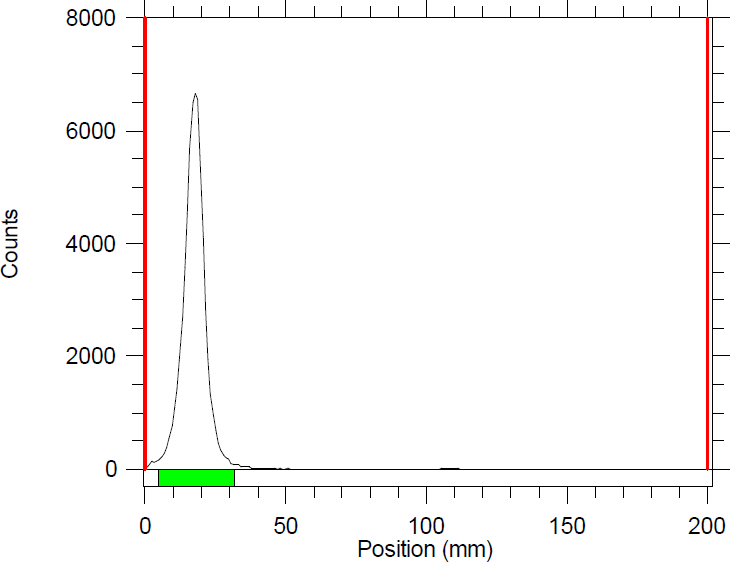


**Figure 1S. Sample radio-TLC results for unlabelled Er-165 ([^165^Er]ErCl_3_) and [^165^Er]Er-PSMA-617 developed using 50 mM EDTA at pH 5.5. Top of the plate is at 110 mm.**

1. **[^165^Er]Er-PSMA-617 Hydrophilicity Results**

Table 2S in Supplementary Information summarizes the hydrophilicity test result across the three replicates. The average LogD_7.4_ value of -2.34 ± 0.24 suggests that the [^165^Er]Er-PSMA-617 radiotracer is highly hydrophilic. LogD_7.4_ result is necessary to predict the excretion pathway of the radiotracer *in vivo*.

**Table 2S. The results of the hydrophilicity tests for [^165^Er]Er-PSMA-617.**

| ID | [^165^Er]Er-PSMA-617 Activity in  n-octanol (kBq) | [^165^Er]Er-PSMA-617 Activity in Water (MBq) | Log D_7.4_ |
| --- | --- | --- | --- |
| Test 1 | 30.2 | 4.03 | -2.13 |
| Test 2 | 10.3 | 3.91 | -2.58 |
| Test 3 | 19.9 | 3.89 | -2.29 |

1. **HPLC Traces**

B

A


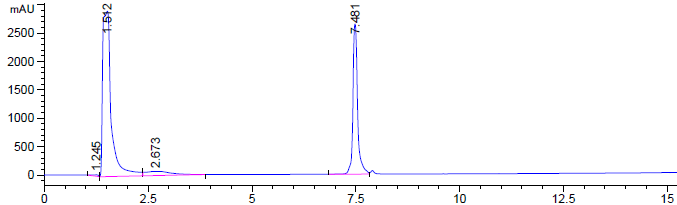

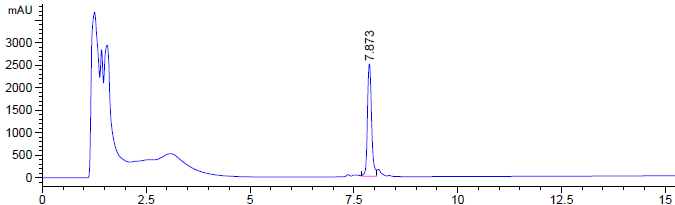


**Figure 2S. The UV (220nm) HPLC traces for A: PSMA-617 and B: [^nat^Er]Er-PSMA-617.**

A

B


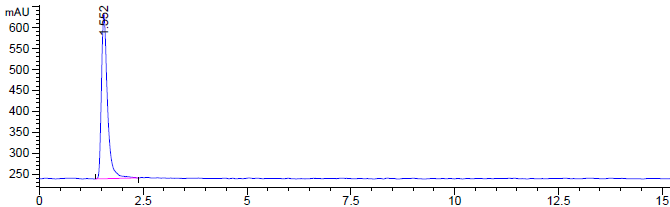

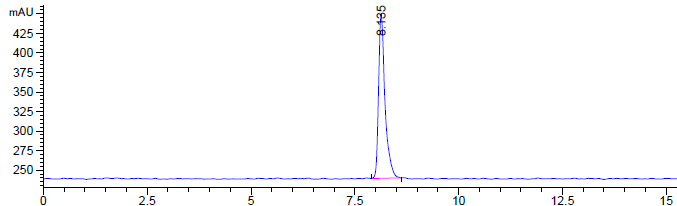


**Figure 3S. The radio-HPLC gamma traces for A: Free ^165^Er and B: [^165^Er]Er-PSMA-617.**

C

B

A


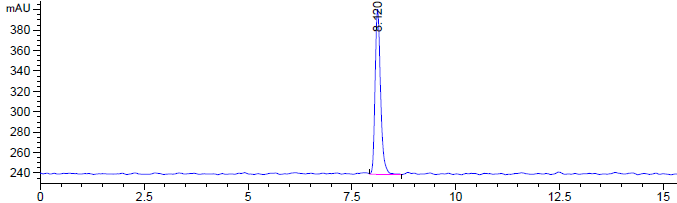

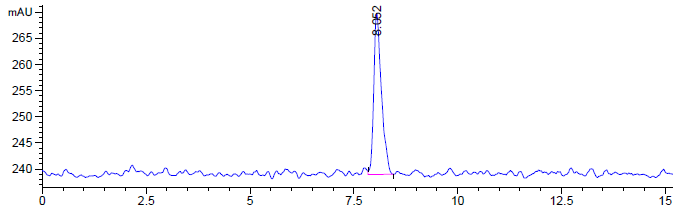

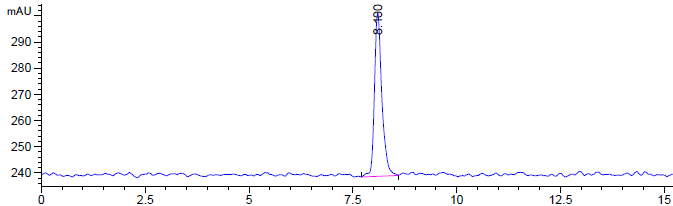


**Figure 4S. The radio-HPLC traces for A: [^165^Er]Er-PSMA-617 incubated in saline, B: [^165^Er]Er-PSMA-617 incubated in mice serum, and C: [^165^Er]Er-PSMA-617 incubated in mice serum for 12 hours.**

1. **Gamma Counter Calibration**

The gamma counter was calibrated using a series of Er-165 activities diluted in serial fashion. The calibration factor was calculated to be 34045 cpm/kBq by fitting a linear curve to the data. This value was used to convert the cpm values obtained from gamma counter for each organ to the corresponding kBq values.

1. **Phantom Imaging**

**Figure 5S. ^165^Er energy spectra acquired by the VECTor scanner for the ^165^Er point source (193.3 MBq/mL). The spectrum on the right focuses on the 0 to 100 keV range.**

1. ***In vivo* [^165^Er]Er-PSMA-617 SPECT/CT Imaging**

The maximum intensity projections (MIP) for other two mice injected with [^165^Er]Er-PSMA-617 that were not shown in the main manuscript in Figure 5 can be seen in Figure 3S.

Mouse 3

Bladder

Kidneys

Tumor

Tumor

Kidneys

Bladder

Mouse 2


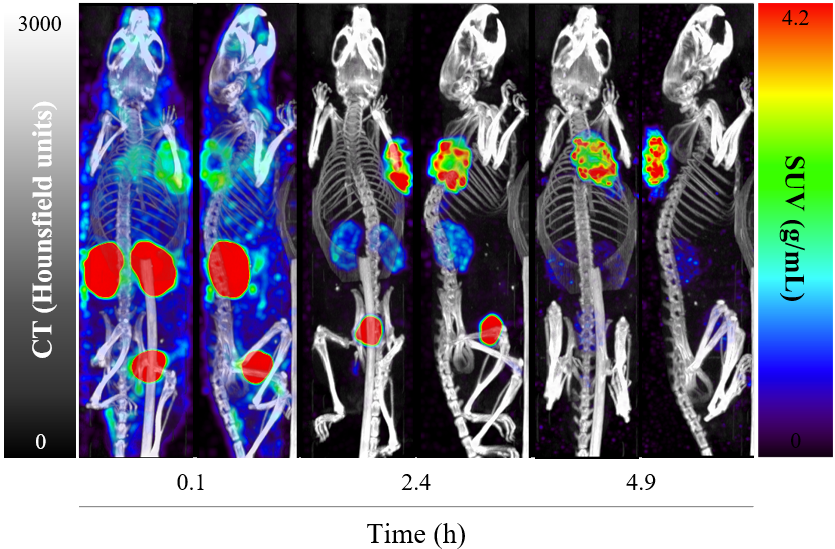

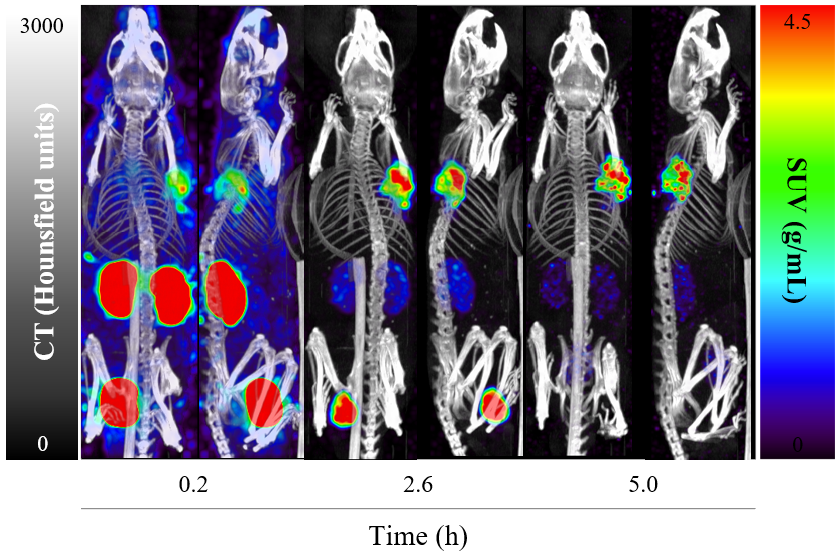


**Figure 6S. Maximum intensity projections (MIP-sagittal and coronal views) from quantitative static SPECT/CT scans recorded at 0.2 h, 2.6 h, and 5.0 h post-administration of [^165^Er]Er-PSMA-617 (n=3).**

1. ***Ex vivo* [^165^Er]ErCl_3_ and [^165^Er]Er-PSMA-617 Full Biodistribution Results**

The full biodistribution results for [^165^Er]ErCl_3_ and [^165^Er]Er-PSMA-617 can be seen in Table 3S. These results are plotted in figures 4 and 5 within the main manuscript.

**Table 3S. The full biodistribution results for LNCaP tumor bearing mice injected with [^165^Er]ErCl_3_ and [^165^Er]Er-PSMA-617 5 hours post-administration.**

| Organ | [^165^Er]ErCl_3_ | [^165^Er]Er-PSMA-617 | |
| --- | --- | --- | --- |
|  | **%IA/g** | **%IA/g** | **SD** |
| blood | 0.63 | 0.01 | 0.00 |
| urine | 21.85 | 13.54 | 20.95 |
| feces | 1.58 | 0.27 | 0.13 |
| brain | 0.04 | 0.01 | 0.00 |
| tumor | 7.06 | 7.86 | 0.46 |
| tail | 10.56 | 0.10 | 0.04 |
| muscle | 0.34 | 0.01 | 0.00 |
| bone | 11.19 | 0.02 | 0.00 |
| bladder | 2.17 | 0.61 | 0.77 |
| pancreas | 0.34 | 0.01 | 0.00 |
| spleen | 3.50 | 0.09 | 0.01 |
| kidneys | 4.87 | 1.50 | 0.38 |
| liver | 4.60 | 0.05 | 0.02 |
| heart | 1.52 | 0.01 | 0.00 |
| lungs | 2.15 | 0.04 | 0.02 |
| stomach | 1.40 | 0.03 | 0.01 |
| small int | 1.17 | 0.04 | 0.03 |
| large int | 0.90 | 0.04 | 0.02 |
| adrenals | 1.12 | 0.08 | 0.02 |
| Testes | 0.38 | 0.03 | 0.01 |
